# Developing an FHIR-Based Computational Pipeline for Automatic Population of Case Report Forms for Colorectal Cancer Clinical Trials Using Electronic Health Records

**DOI:** 10.1200/CCI.19.00116

**Published:** 2020-03-05

**Authors:** Nansu Zong, Andrew Wen, Daniel J. Stone, Deepak K. Sharma, Chen Wang, Yue Yu, Hongfang Liu, Qian Shi, Guoqian Jiang

**Affiliations:** ^1^ Department of Health Sciences Research, Mayo Clinic, Rochester, MN

## Abstract

**PURPOSE:**

The Fast Healthcare Interoperability Resources (FHIR) is emerging as a next-generation standards framework developed by HL7 for exchanging electronic health care data. The modeling capability of FHIR in standardizing cancer data has been gaining increasing attention by the cancer research informatics community. However, few studies have been conducted to examine the capability of FHIR in electronic data capture (EDC) applications for effective cancer clinical trials. The objective of this study was to design, develop, and evaluate an FHIR-based method that enables the automation of the case report forms (CRFs) population for cancer clinical trials using real-world electronic health records (EHRs).

**MATERIALS AND METHODS:**

We developed an FHIR-based computational pipeline of EDC with a case study for modeling colorectal cancer trials. We first leveraged an existing FHIR-based cancer profile to represent EHR data of patients with colorectal cancer, and then we used the FHIR Questionnaire and QuestionnaireResponse resources to represent the CRFs and their data population. To test the accuracy of and overall quality of the computational pipeline, we used synoptic reports of 287 Mayo Clinic patients with colorectal cancer from 2013 to 2019 with standard measures of precision, recall, and F1 score.

**RESULTS:**

Using the computational pipeline, a total of 1,037 synoptic reports were successfully converted as the instances of the FHIR-based cancer profile. The average accuracy for converting all data elements (excluding tumor perforation) of the cancer profile was 0.99, using 200 randomly selected records. The average F1 score for populating nine questions of the CRFs in a real-world colorectal cancer trial was 0.95, using 100 randomly selected records.

**CONCLUSION:**

We demonstrated that it is feasible to populate CRFs with EHR data in an automated manner with satisfactory performance. The outcome of the study provides helpful insight into future directions in implementing FHIR-based EDC applications for modern cancer clinical trials.

## INTRODUCTION

It has been increasingly recognized that the common data collection and management methods used by the oncology clinical trial community are laborious, imprecise, and expensive, which greatly hinders the implementation of novel trial design and the achievement of study integrity and reproducibility for trial. For example, rapid data inputs are required for adaptive design, especially when the adaptation of the trial depends on accurate individual patients’ outcome data status, which can be obtained quickly. The case report forms (CRFs) are questionnaires specifically used by researchers in clinical trial research to collect information about each participating patient.^1^ The development and population of the CRFs play a significant role in the selection of participants.^2^ One trend is to design the electronic data capture (EDC)-oriented electronic forms using model-driven solutions, such as FHIRForm,^3^ Research Electronic Data Capture,^4^ and OpenClinica.^5^ However, the rapid growth in the scale of medical data and collaborations between different medical facilities for developing clinical trials creates new challenges. These challenges include integrating data across different EHR systems as well as ensuring the data required by the protocol and study-specific hypothesis is attributed in an efficient way.^4,6,7^ There is a benefit of having a standard for the format as well as a clear value definition for data and responses of CRFs to facilitate EDC, which allows plug and play functionality for any developed CRFs, because they will be interuseable between the different institutions as long as data are generated in that standardized format.^8,9^

There are a few efforts to provide a standardized data model for the secondary use of electronic health record (EHR) data, such as Informatics for Integrating Biology and the Bedside,^10^ the Observational Health Data Sciences and Informatics Common Data Model,^11^ and Fast Healthcare Interoperability Resources (FHIR).^12^ Notably among these, FHIR is emerging as the next-generation standards framework for exchanging electronic health care data. FHIR defines data formats and elements, known as resources, as well as messages to exchange medical records. FHIR provides a standard data communication method that directly delivers discrete data elements, such as Patient, Diagnosis, Procedure, and Medication, rather than the traditional document-centric methods, and enables data to be quickly transitioned and easily parsed by analytics platforms.^8,12^ The importance of standardization for cancer phenotypic data has been increasingly recognized by the cancer research informatics community. For example, the Clinical Data Interchange Standards Consortium has published a number of therapeutic area standards for cancers.^13^ The Royal College of Pathologists of Australasia (RCPA)/HL7 Australia^14^ has released the cancer profiles for structured colorectal and prostate reports.

**CONTEXT**

**Key Objective**
The objective of this study was to examine the existing cancer model based on a design of pipeline to harmonize with real-world electronic health records (EHRs) for the automation of the case report forms (CRFs) population for cancer clinical trials.
**Knowledge Generated**
We demonstrated it is feasible to populate CRFs with Fast Healthcare Interoperability Resources (FHIR)-based EHR data in an automated manner with high performance. We observed limited information loss in the extract, transform, load process to generate a standard-based pathology-report data representation, because there was a similar performance for the CRF population with the standardized representation versus raw pathology data.
**Relevance**
With the FHIR-based CRF population pipeline prototyped in this study, the data collection, transformation, and quality assurance process became streamlined and generalizable to support further adaptation of other cancer types.

To meet the EDC requirements for cancer clinical trials, the existing models need to be carefully examined and harmonized with real-world EHR and cancer trial data. In particular, the examination of the capability of FHIR in EDC applications for effective cancer clinical trials needed to be conducted. The objective of the study was to design, develop, and evaluate an FHIR-based method that enables the automation of the CRF population for cancer clinical trials using real-world EHRs. As such, we developed a computational pipeline with two corresponding efforts conducted: (1) FHIR-based data representation for Mayo Clinic patients with colorectal cancer, based on an existing cancer profile, the Australian Colorectal Cancer Profile^14^; and (2) FHIR-based CRFs’ representation and population. As a proof of concept, we conducted a case study for modeling cancer phenotypic data from Mayo Clinic pathologic reports and populated the CRFs designed for colorectal cancer trials.^15^

## MATERIALS AND METHODS

### Data

Mayo Clinic’s Unified Data Platform (UDP)^16^ is a clinical data warehouse that provides a combined view of heterogeneous data across multiple data sources, including Epic Clarity, through effective data orchestration. UDP provides access to all the information on patients with colorectal cancer. In practice, we only extract the surgical and pathologic information in this study. For surgical information, we collected surgical reports for obtaining cancer-specific data required by clinical trials. For pathologic information, we mainly used a semistructuralized form known as a synoptic report, as well as the original pathologic report as a supplementary source. A synoptic report is an internal effort by the Mayo Clinic since 2013 to enforce compliance with the College of American Pathologists’ protocols^17^ on exactly what data elements must be included and generally have roughly templated values^18^ when documenting certain cancers within pathology reports. The protocol of clinical data access was approved by the Mayo Clinic Institutional Review Board.

### FHIR-Based Standardization and Tools

The resources defined by FHIR cover a wide range of concepts that are clinically related (eg, Clinical) and supportive (eg, Foundation, Base, Financial, and Specialized). Recognized as classes, those concepts and their subconcepts (ie, subclasses) can better interpret and facilitate the use of resources. For example, the two resources *Observation* and *DiagnosticReport* belong to the subconcept *Diagnostic* under the concept *Clinical* to classify the resources. To model the EHR data, a resource is described with a set of attributes. Each attribute is limited to be valued with certain predefined data types, such as “*string*,” “*dateTime*,” and “*Reference*,” “*CodeableConcepts*,” and “*code*.” The popular clinically related terminologies, such as SNOMED CT,^19^ Logical Observation Identifiers Names and Codes (LOINC),^20^ and International Classification of Diseases–9 and –10,^21^ are adopted as preferred vocabularies.

HL7 application programming interface (HAPI)-FHIR^22^ is an open-source Java library implementation of the FHIR specification for data modeling, parsing, and management. HAPI-FHIR supports the following tools: (1) parser and encoder to convert between the source data model and FHIR-based data model, and (2) communicate between the client application and server.

### Australian Colorectal Cancer Profile

RCPA has initiated an effort for adopting the use of structured cancer reporting and the use of FHIR to facilitate the data exchange.^14^ As mentioned, RCPA has released the cancer profiles for structured colorectal and prostate reports. The colorectal cancer protocol used in this study is based on the structured colorectal cancer profile released by RCPA.^23^ A logical model that captures the concepts and defines the value sets for the published protocol is formed and is further be represented with FHIR. The atomic data items in the report are mainly represented as the FHIR resources *DiagnosticReport* and *Observation*.

### Automatic Population of CRFs

**FIG 1.**
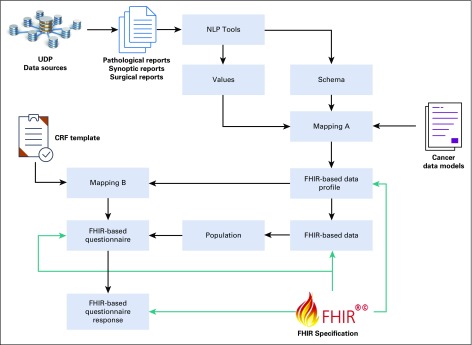
A Fast Healthcare Interoperability Resources (FHIR)–based pipeline for automatic data population of CRFs. CRF, case report form; NLP, natural language processing; UDP, Unified Data Platform.

We proposed an FHIR-based method that enables the standard representation of data elements from the pathologic report for cancer trials and functionalizes the automation of the data population of CRFs to support real-world cancer trials. There are three main steps, as shown in Figure 1: (1) cancer data preparation, (2) FHIR-based data profiling, and (3) FHIR-based CRF data population.

First, pathologic and surgical reports are extracted from UDP. The original unstructured reports are converted to structured reports that better describe the data with the schema (ie, data elements) by natural language processing (NLP) tools. To allow this study to focus primarily on the automated data population of CRFs in an interoperable manner, in practice, instead of directly applying NLP tools to obtain the structured information from pathologic reports, we used the semistructured synoptic reports that represent the pathologic reports with a structured presentation. From the synoptic reports, a total of 21 data elements, such as tumor site, tumor size, and surgical margins, are directly extracted and used to populate the data model. For the data elements that cannot be covered by synoptic reports, we also use the original pathologic reports to complement the capture of additional information based on simple NLP-based methods. Similarly, we applied the same NLP-based methods for pathologic reports to process surgical reports.

Second, the Australian Colorectal Cancer Profile (ACP) was used as the data model to describe and standardize the data extracted from the pathologic reports. In practice, a small subset of 21 data elements from ACP was extracted to map the element from the synoptic report manually. For example, the element tumor site from the synoptic report is mapped to *Colorectal.preAnalytic.tumourLocation* and *Colorectal.macro.tumourSite*. The complete list of mapping is presented in Table 1. We adopted the mappings of data elements between ACP and FHIR (defined in the ACP) to enable the FHIR-based representation of data. For example, *Colorectal.macro.tumourSite* can be represented with the FHIR resource *Observation* with the value *LOINC#33725-3 (Tumor site).* The detailed mapping table and conversion script can be found at http://hl7.org.au/fhir/rcpa/cmap.html#summary. The data conversion was based on standard string processing, such as tokenization, stemming (ie, lemmatization), and dictionary look-up to represent the raw values with standardized terminology.

**TABLE 1.**
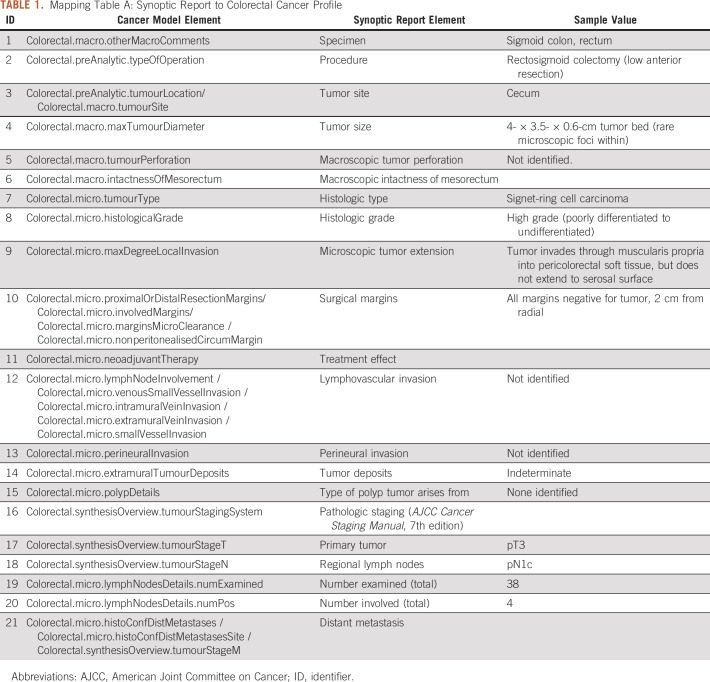
Mapping Table A: Synoptic Report to Colorectal Cancer Profile

**FIG 2.**
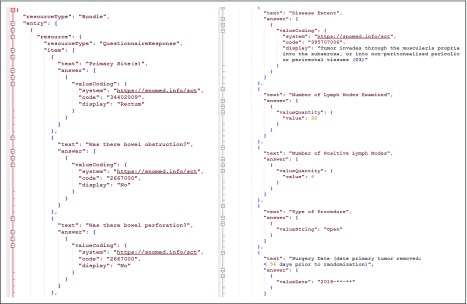
An example of the Fast Healthcare Interoperability Resources–based case report form populated with data.

Last, in step 3, a CRF is represented with an FHIR resource *Questionnaire,* and the answers from each patient are generated via a mapping between the question items and the data elements of ACP. For example, “Primary Site(s) of a patient” is mapped to *Colorectal.preAnalytic.tumourLocation* and *Colorectal.macro.tumourSite.* The detailed mappings for nine questions are listed in Table 2. Note that, for questions 7 and 8 regarding the procedure information of a patient, we extended the ACP with an extension defined in the FHIR resource *Observation* to represent procedure information. We used the FHIR resource “*QuestionnaireResponse”* to represent answers to the questions in the CRF, as shown in our example in Figure 2.

**TABLE 2.**
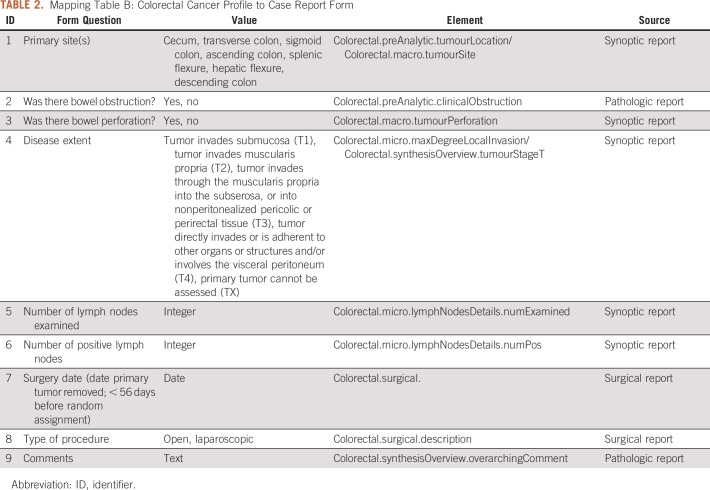
Mapping Table B: Colorectal Cancer Profile to Case Report Form

### Creation of Mappings

We created two sets of mappings, as shown in Tables 1 and 2: mapping A and mapping B.

Mapping A (Table 1) provides a map for the information from the structure of the synoptic report to the ACP. For example, pathologic information, such as tumor site and microscopic tumor extension, is obtained from a synoptic report and is mapped to the corresponding element in ACP. Mapping B (Table 2) provides a map from the ACP FHIR model to the CRF questions, which is used to populate the CRFs represented by FHIR “*QuestionnaireResponse*.” In this study, we used the colorectal cancer adjuvant on a study form used in the study by Alberts et al,^15^ which contains nine primary questions: “Q1 Primary Site(s),” “Q2 Was there bowel obstruction,” “Q3 Was there bowel perforation,” “Q4 Disease extent,” “Q5 Number of lymph nodes examined,” “Q6 Number of positive lymph nodes,” “Q7 Surgery date,” “Q8 Type of procedure,” and “Q9 Comments.” Please note, to completely populate the CRF with data, we also used the original pathologic reports and surgical reports as the supplementary data sources for the corresponding mappings to address the four questions, Q2, Q7, Q8, and Q9. Because the values for the answers are restricted, such as “Yes” for “Was there bowel obstruction” and “Open/ Laparoscopic” for “Type of procedure,” we were able to design an extraction strategy based on the simple rules to obtain the answers.

### Experiments

To test our pipeline, we extracted 1,037 colorectal cancer synoptic reports of 287 Mayo Clinic patients from UDP dated from 2013 to 2019. We obtained the data from a UDP search based on the colorectal cancer–related International Classification of Diseases–9 codes filtered in compliance with Mayo Clinic’s policies of research authorization. We ran our pipeline successfully against 1,037 synoptic reports and populated them into the FHIR-based cancer profile instances. Our pipeline further populated the FHIR Questionnaire resource (represents the answers to the CRF) with data points from both the FHIR cancer profile instances and structured data.

Figure 2 shows an example of a populated FHIR-based CRF. The form is organized with FHIR resource *Bundle*, which contains an FHIR resource *QuestionnaireResponse* that represents the responses as the entry. Each question in CRF is considered a question item, which includes the original question and the filled answers. For example, Q1 with an answer “Rectum” is represented in the first item. The values are represented by the standardized values of coding systems. In the same example, “Rectum” is encoded as “34402009” from SNOMED CT.^19^ We applied diverse data formats to support the presentation of values, such as quantity (eg, “Q6”), string (eg, “Q8”), date (eg, “Q7”) and text (eg, “Q9”).

We designed two tasks to evaluate the accuracy of the ACP conversion in the module and the overall quality of the automatically populated CRFs.

#### Task 1: ACP-based data conversion.

We randomly selected 200 records, with any sensitive information removed that could be used to identify the patient, to evaluate how precisely the values of all the data elements are converted. The results were reviewed by two subject matter experts (N.Z. and G.J.) majoring in medical informatics and metadata harmonization. The κ score of the two experts for interagreement was 0.90. The reviewers marked the true positive (TP), true negative (TN), false positive (FP), false negative (FN) defined as follows:

- If the value is “present” and the pipeline correctly parsed it, we labeled it as TP.
- If the value is “present” and the pipeline wrongly parsed it, we labeled it as FN.
- If the value is “absent” and the pipeline correctly recognized it, we labeled it as TN.
- If the value is “absent” and the pipeline wrongly asserted a value, we labeled it as FP.

Then, the accuracy of conversion could be evaluated for each element on the basis of the counts of those measures by reviewers.

#### Task 2: CRF data population

We randomly selected 100 deidentified records to evaluate the overall quality of the populated CRFs. The results were reviewed by two medical informatics experts (Y.Y. and G.J.); the interagreement κ score was 0.97. The reviewers were required to complete the answers for each question on the basis of an investigation of the given original synoptic reports. The answers annotated by the reviewers were considered the gold standard, and the precision, recall, and F1 score were calculated correspondingly.

## RESULTS

### Task 1: ACP-Based Data Conversion

As Table 3 shows, most of the values mapped perfectly to the corresponding concepts as defined in the cancer profile for all the elements (average accuracy, 0.99) when tumor perforation was excluded (0.09). Looking into the reasons for the low performance of tumor perforation, we found that a mapping rule was missing for the values of the tumor perforation, resulting in most of the FNs recorded. Other FNs were caused by unexpected data formats or data values, such as a one-dimensional value instead of three-dimensional values for *maxTumourDiameter* or *ileum*, *rectosigmoid* colon used for tumor site.

**TABLE 3.**
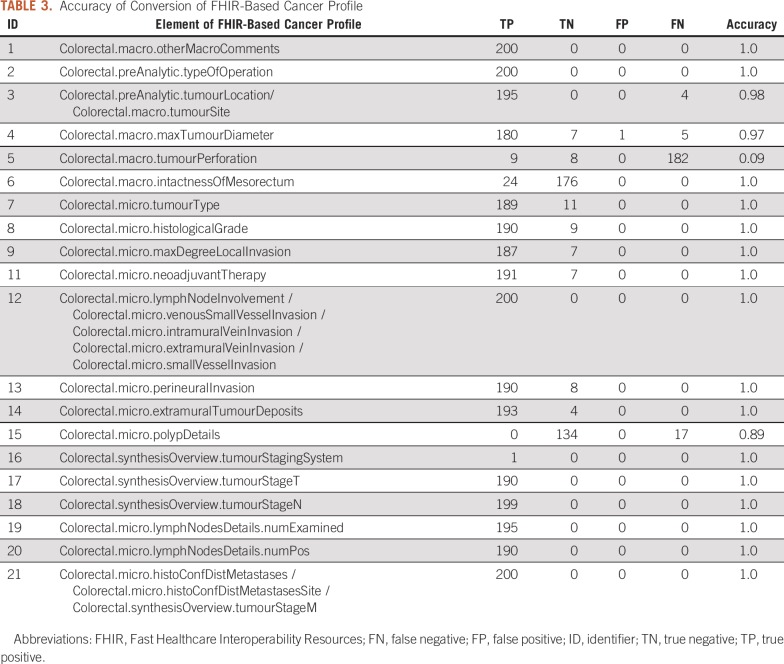
Accuracy of Conversion of FHIR-Based Cancer Profile

### Task 2: CRF Data Population

As Table 4 shows, those elements populated on the basis of the source of structured sources, such as surgical date and type, and bowel obstruction, received perfect precision (1.0), recall (1.0), and F1-score (1.0) values. The elements populated on the basis of the synoptic report received the average F1 score of 0.95.

**TABLE 4.**
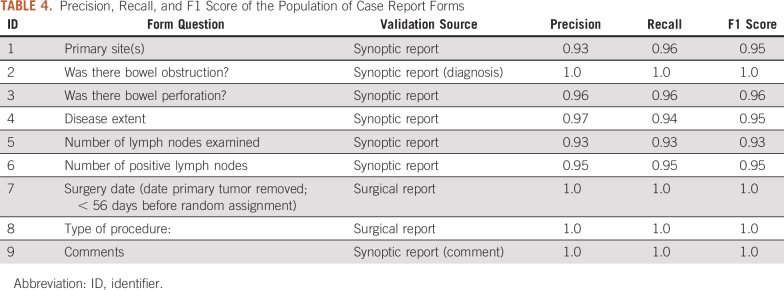
Precision, Recall, and F1 Score of the Population of Case Report Forms

## DISCUSSION

In this study, we used the FHIR standard as a unified framework for automating the CRFs data population for cancer clinical trials. We performed a case study and implemented a computational pipeline focusing on the population of an on-study CRF form for a colorectal cancer trial. We demonstrated that it is feasible to populate CRFs with EHR data in an automated manner, as can be seen by the high performance shown in Table 4, based on the adoption of the FHIR as a standard data access mechanism. Please note, the purpose of the experiment and the collected performance metrics is to demonstrate the reliability of our proposed method for automatically generating the CRFs from raw data. Because the mappings and NLP-based string processing play an important role, despite the performance metric being intended to cover our whole pipeline, the results were influenced by the performance of the NLP methods used. The reason for choosing FHIR can be summarized as follows: (1) FHIR is one of the most popular data standards in the medical field, with all major EHRs adopting FHIR for health care data exchange; and (2) FHIR is a messaging standard that allows information to be captured as it is generated. As opposed to the Observational Health Data Sciences and Informatics Common Data Model and Integrating Biology and the Bedside, which typically are the secondary use of EHR data requiring batched queries, the mechanism of FHIR makes it easier to populate the data model and keep it updated. With the FHIR-based CRF population pipeline prototyped in this study, the data collection, transformation, and quality assurance process became streamlined and can be replaced.

Despite the reliability of the proposed framework, as demonstrated in this study, there are a number of limitations in this study. First, we just used a single on-study CRF with limited data elements included. Although this study is a proof of concept, a single CRF is certainly not enough to evaluate the coverage of the existing cancer profiles. In the future, we will look into more CRFs to fully understand the data element requirements that should be able to inform the enhancement of cancer profile development. Second, we adopted the ACP to cover most of the elements from synoptic reports. However, our target CRF asks for more data elements than those covered. We argue that in the future, a cancer profile with a comprehensive list of data elements from both structured and unstructured data should be developed. Third, most of the cancer-specific phenotypic data are largely embedded in the unstructured clinical narratives. In this study, we used the synoptic reports Mayo Clinic has implemented.^17^ Although we have argued that the use of synoptic reports can greatly reduce the complexity for the pipeline implementation, as well as cover many high-quality data elements, we realize the necessity of using advanced NLP tools, because the structured or semistructured reports (eg, synoptic reports) are not always available. We have developed an FHIR-based clinical-data normalization tool that enables the extraction of structured information from unstructured medical notes,^24,25^ which can be adapted. In addition, more sophisticated tools like MedKAT^26^ and DeepPhe^27^ need to be examined for processing the narratives in diverse notes to extend the coverage of more questions. Last, the mapping rules and evaluation methods developed in this study were based on the consensus from our study team and done on a small scale as a proof of concept. In the future, a more sophisticated and rigorous method and evaluation (eg, a community-based consensus) will need to be designed and conducted.
